# LncRNAs: Proverbial Genomic “Junk” or Key Epigenetic Regulators During Cardiac Fibrosis in Diabetes?

**DOI:** 10.3389/fcvm.2018.00028

**Published:** 2018-04-04

**Authors:** Saumik Biswas, Anu Alice Thomas, Subrata Chakrabarti

**Affiliations:** ^1^Department of Pathology and Laboratory Medicine, Western University, London, ON, Canada

**Keywords:** diabetic cardiomyopathy, epigenetics, long non-coding RNAs, cardiac fibrosis, EndMT

## Abstract

Long non-coding RNAs (lncRNAs) are critical regulators in a multitude of biological processes. Recent evidences demonstrate potential pathogenetic implications of lncRNAs in diabetic cardiomyopathy (DCM); however, the majority of lncRNAs have not been comprehensively characterized. While the precise molecular mechanisms underlying the functions of lncRNAs remain to be deciphered in DCM, emerging data in other pathophysiological conditions suggests that lncRNAs can have versatile features such as genomic imprinting, acting as guides for certain histone-modifying complexes, serving as scaffolds for specific molecules, or acting as molecular sponges. In an effort to better understand these features of lncRNAs in the context of DCM, our review will first summarize some of the key molecular alterations that occur during fibrosis in the diabetic heart (extracellular proteins and endothelial-to-mesenchymal transitioning), followed by a review of the current knowledge on the crosstalk between lncRNAs and major epigenetic mechanisms (histone methylation, histone acetylation, DNA methylation, and microRNAs) within this fibrotic process.

## Introduction

With the incidence of diabetic cardiomyopathy (DCM) increasing at an alarming rate, the need for broadening the therapeutic scope for disease management becomes fundamental. In order to develop new therapeutic agents to successfully impede the progression of DCM, a thorough understanding of the complex pathogenetic mechanisms implicated in DCM progression is an absolute requirement. There are currently several known metabolic pathway alterations that occur in a hyperglycemic environment; however, recent advances in genomic technology have identified that a significant number of epigenetic alterations contribute to the development and progression of DCM. Long non-coding RNAs (lncRNAs), which are involved in altering gene expression without modifying the underlying nucleotide composition of the genome, are beginning to emerge as key epigenetic regulators in various diseases (1). Despite possessing limited protein-coding potential and being greater than 200 nucleotides in length, lncRNAs can alter the dynamic configuration of the chromatin by interacting with several enzymes that facilitate chromatin remodeling and gene regulation (2). In the context of DCM, numerous lncRNAs have been identified to be aberrantly expressed in diabetic cardiac tissues (3); however, the majority of lncRNAs have not been comprehensively characterized (4). To fill some of the gap in knowledge, this review will first provide the necessary background behind DCM, extracellular matrix (ECM) proteins, and fibrosis and then discuss the postulated roles of lncRNAs in major epigenetic modifications during DCM fibrosis.

## Diabetic Cardiomyopathy (DCM)

Cardiovascular complications are responsible for the majority of diabetes-related morbidity and mortality (5). Such complications include atherosclerosis, autonomic neuropathy and diabetic cardiomyopathy (DCM). The latter leads to an increased risk for the development of structural and functional changes in the myocardium independent of coronary artery disease and hypertension (6). Rubler and colleagues first described this ventricular dysfunction occurring in diabetic patients in 1972 (7). Alterations in myocardial structure, calcium signalling and metabolism are primary signs that precede accelerated left ventricular hypertrophy and increase susceptibility to ischemic injury and overall heart failure (HF) (8). The myocardial tissue undergoes structural and functional modifications after diabetes, which is induced by hyperglycemia, hyperlipidemia and insulin resistance (9). A histological trademark of DCM is interstitial and perivascular fibrosis, characterized by increased deposition of collagen accompanied by crosslinking of these collagen fibers contributing to reduced ventricular compliance (7, 10, 11). Myocardial fibrosis is accompanied by an increase in left ventricle (LV) mass, also known as left ventricle hypertrophy (LVH). LVH has been linked to elevated markers of systemic inflammation such as fibrinogen, C-reactive protein and microalbuminuria (12). These structural changes are accompanied by functional alterations, namely diastolic and systolic dysfunction. Diastolic dysfunction is defined as defective ventricular relaxation leading to pressure increase and impairment in blood filling during diastole (9). During systolic dysfunction, the myocardium fails to eject adequate blood volume and is observed at later stages in DCM after diastolic dysfunction has been established in patients (13, 14). Early identification of these abnormalities is important to provide appropriate treatment and prevent advancement to HF.

Multiple mechanisms have been proposed to explain the pathogenesis of DCM. These include autonomic dysfunction, defects in lipid metabolism, abnormalities in ion homeostasis, alterations in structural proteins, increased oxidative stress, interstitial fibrosis and alterations in myocardial substrates and energy metabolism (15–20). As compensation for glucose assimilation, fatty acid transporters are increased to generate ATP through fatty acid (FA) degradation (19). However, excess FAs accumulate in the cytosol and cause lipotoxicity through the generation of diacylglycerol and ROS. Hyperglycemia also triggers ROS and advanced glycation end-product (AGE) production, culminating in cardiac glucotoxicity. Hence, the lack of fuel and the presence of lipotoxicity and glucotoxicity trigger cardiac inflammation, fibrosis and contractile dysfunction (21). In response, RAS and TGF-β systems that mediate cytokine/chemokine responses are significantly enhanced (22).

Currently, there is no single therapy for treating DCM. Treatment options revolve around dietary glycemic control, direct and indirect regulators of fatty acid metabolism, and inhibitors of factors that trigger heart failure symptoms (13, 23). Medical advancements and lifestyle interventions have contributed to reduction in cardiovascular mortality in diabetic patients. However, epidemiological studies show higher incidence of diabetic cardiomyopathy despite adjustments for hypertension, microvascular diseases, hypercholesterolemia, body mass index and other factors (24–26). Therefore, further insights into the pathological mechanisms behind the advancement of DCM are warranted and the characterization of these processes may open novel avenues for targeted therapies. Since increased extracellular matrix (ECM) protein deposition or cardiac fibrosis is a key event of DCM, we chose to focus on this area.

## Extracellular Matrix (ECM) Proteins

**Figure 1:**
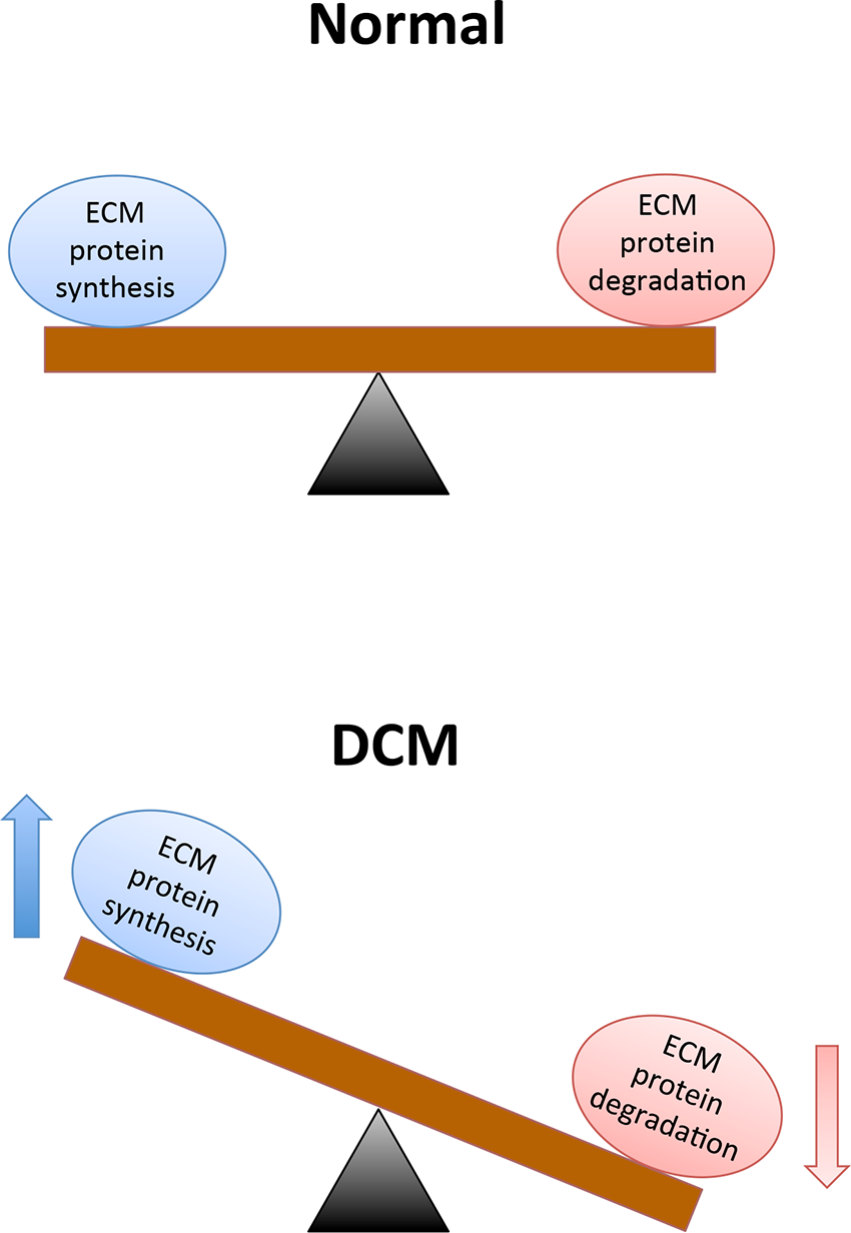
A chronic hyperglycemic environment causes excessive extracellular matrix protein deposition, which subsequently contributes to cardiac fibrosis and cardiac dysfunction. Legend: ECM = Extracellular matrix, and DCM = Diabetic cardiomyopathy.

Structural and functional alterations in the vasculature arise in the presence of chronic diabetes. Among these alterations, modifications to the extracellular matrix (ECM) and basement membrane (BM) are the structural hallmarks in target organs of diabetic complications (27). First recorded by Siperstein and colleagues in 1968, the disturbances of ECM are directly linked functional loss in target organs (28). ECM encompasses an insoluble network of collagens, fibronectin, elastins, structural glycoproteins, proteoglycan hyaluronans and integrins that provide cells with mechanical support and mediate multifarious interactions between other cells or the ECM of vascular tissues (29). The cardiac ECM consists of fibrillar collagen localized within myocardial interstitium and non-fibrillary collagen, as well as fibronectin and laminin in the myocyte basement membrane (30, 31). In the context of DCM, chronic hyperglycemic environments can initiate a cascade of signals that disrupt the balance between synthesis and breakdown of ECM components (shown in Figure 1); which, ultimately contributes to the development of LVH and hypertension-induced diastolic dysfunction (32–37). Prior to the development of cardiac dysfunction, excessive ECM protein deposition occurs in the heart muscles, accompanied by abnormal proliferation of cardiac fibroblasts, and this phenomenon is known as cardiac fibrosis (38). Cardiac fibrosis plays a critical role in the development of DCM and TGF-ß is one of the most studied mediators of this phenomenon (39, 40). Numerous studies have reported high-glucose mediated elevation in the transcription of TGF-ß genes, thereby increasing the levels of the protein and its downstream signaling (41, 42). In addition to its role in the canonical SMAD signaling pathway, TGF-ß is also the key stimulator of three known MAP pathways: ERK, JNK and p38 pathways (43–45). Evidently, TGF-ß is the chief cytokine in the regulation of ECM protein synthesis and is responsible for stimulating the production of its components including proteoglycans, fibronectin and collagen, while blocking matrix degradation (46). Moreover, in addition to TGF-ß, hyperglycemia-induced damage of endothelial cells (ECs) can activate vascular endothelial growth factor (VEGF)-mediated angiogenic responses that may further contribute to increased basement membrane (BM) thickening and ECM protein deposition (47–49).

## Endothelial-Mesenchymal Transition (EndMT)

**Figure 2:**
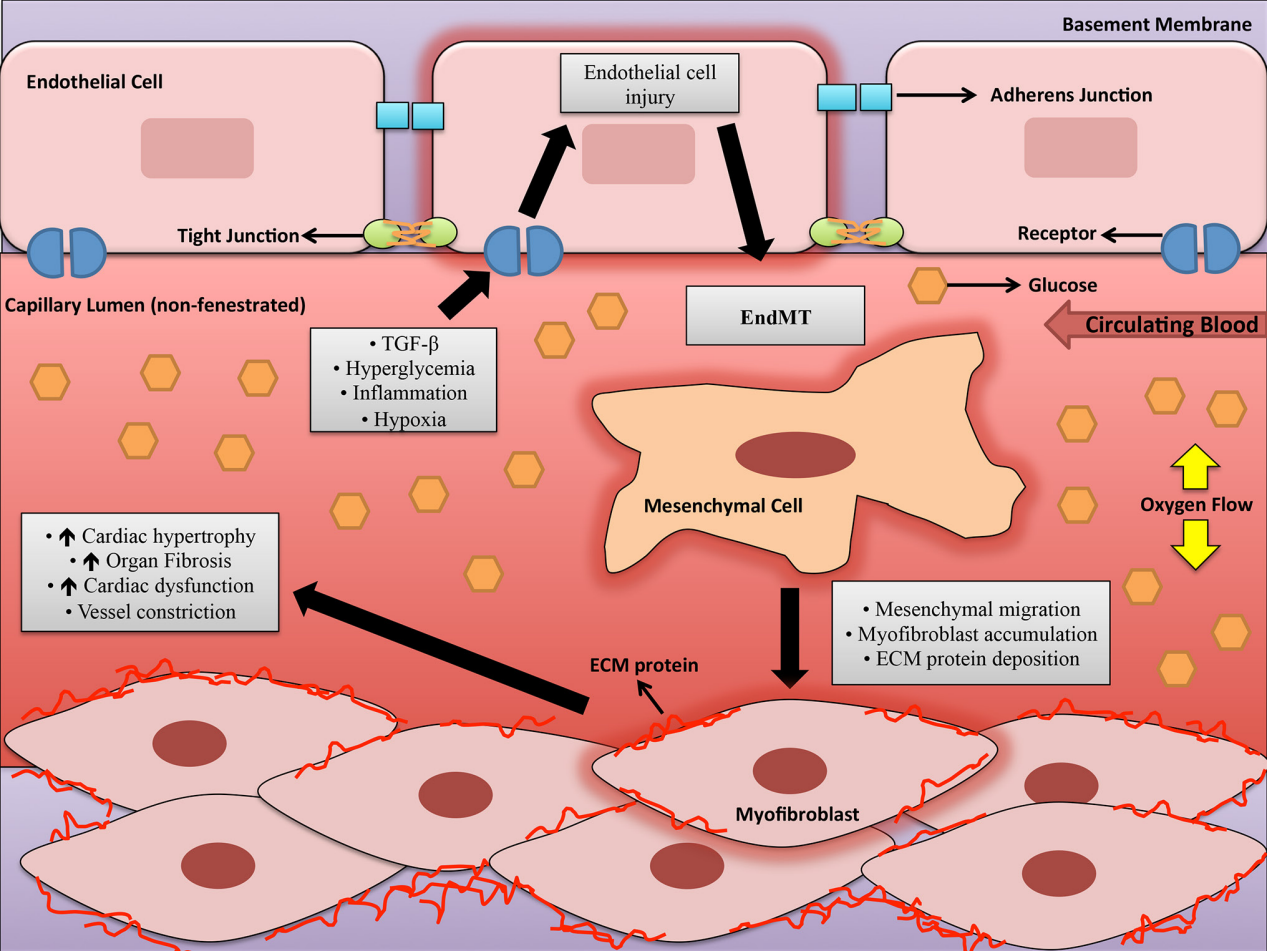
A schematic depicting some of the factors involved in endothelial-to-mesenchymal transition in the capillary lumen during diabetic cardiomyopathy. Legend: ECM = Extracellular matrix, EndMT = Endothelial to-mesenchymal transition, and TGF-β=Transforming growth factor-beta.

The entire circulatory system is lined by ECs, forming a boundary between circulating blood in the lumen and all vessel walls from the heart to small capillaries (50). ECs allow the blood to be pumped further by reducing turbulence of flow and are unambiguously known to play a critical role in maintaining overall homeostasis (50, 51). The endothelium secretes a number of factors that regulate coagulation, fibrinolysis, platelet aggregation and vascular tone (51). Hyperglycemia causes the endothelium to be exposed to a range of negative intracellular occurrences that promote endothelial dysfunction, where the endothelium loses its physiological properties (51). Fibrosis, or accumulation of fibrous connective tissue, and excess ECM in and around inflamed or damaged tissue that culminates in organ failure or death is a characteristic feature of endothelial dysfunction (52–55). Fibrosis involves proliferation of local fibroblasts and their differentiation into myofibroblasts (55). Myofibroblasts, in comparison to fibroblasts, have elevated α-smooth muscle actin (α-SMA) and an upregulated production of ECM proteins like type IV collagens (55, 56). Initially, the origin of these myofibroblasts were thought to be from local proliferating resident fibroblasts in response to factors like TGF-ß, but subsequent research suggests other cellular sources such as ECs that can form myofibroblasts (57, 58). ECs can adopt a mesenchymal phenotype and express markers characteristic of myofibroblast differentiation, which can include α-SMA, smooth muscle 22α (SM22α), vimentin, fibroblast-specific protein 1 (FSP1) and ECM proteins like fibronectin (FN) and collagen. As well, EC markers such as vascular endothelial cadherin (VE-Cadherin) and cluster of differentiation 31 (CD-31) are downregulated in these mesenchymal-like ECs (58–60). ECs undergo this phenomenon known as endothelial-mesenchymal transition (EndMT) to gain an altered differentiated phenotype and obtain invasive and migratory abilities in order to affect pathological processes in different ways (shown in Figure 2) (50). EndMT can be defined as loss of cell adhesion and actin reorganization to convert apical-basal polarity to front-end/back-end polarity resulting in change from compact, well-structured cobblestone-like shape to less organized spindle-shaped cells (60, 61). The major regulatory cytokines that stimulate EndMT are the TGF-ß superfamily of proteins including TGF-ß1 and TGF-ß2 (58, 61–63). Importance of this major regulator in the activation of EndMT has been described previously (64). TGF promotes EndMT through Smad-dependent and Smad-independent pathways like protein kinase C (PKC) (62, 65). Moreover, several studies have shown that inhibition at TGF-signaling at different stages have reduced EndMT and fibrosis in animal models (62, 64, 66). TGF-signaling play important roles in myofibroblast differentiation and ECM alterations, which favor myofibroblast transdifferentiation through altered responses to mechanical stress or transduction of growth factor signals (58). TGF is responsible for the induction of cardiac fibroblast transdifferentiation to myofibroblasts and promotes cardiac fibrosis by inducing ECM protein synthesis and reducing collagenase expression (67, 68). In general, TGF-signaling and EndMT are major contributors to generation of myofibroblasts that are key role players in the development of fibrotic diseases such as DCM.

## The Emergence of Epigenetics

 Genetic factors are thought to be important for the development of diabetes and its various complications. In addition to genetics, environmental factors such as sedentary lifestyle, age and obesity are important for elevating the risk for the disease. In fact, there is considerable evidence indicating that the interaction between genes and the environment can influence an individual’s susceptibility to develop a chronic complication (69–71). The environmental factors can alter signaling pathways and alter gene expression through epigenetic modifications. Epigenetics can be defined as the study of heritable changes in gene expression that does not involve changes to the underlying DNA sequence. This represents a change in phenotype without a change in genotype that affects how cells read genes (72). The chromosomal DNA is tightly packaged into chromatin, and its status between transcriptionally “active” (euchromatin) and “inactive” (hetero-chromatin) in response to extracellular signals is a key aspect that can govern the expression of genes (73–75). The chromatin is composed of subunits known as nucleosomes. Each nucleosome has an octamer of histones, with two copies of histone proteins (H2A, H2B, H3 and H4), wrapped by 147 base pairs of chromosomal DNA. Post-translational modifications of these histones (PTHMs) alter their interactions with DNA and represent one of the key epigenetic regulations (76, 77). Epigenetic regulations can lead to non-heritable or heritable effects. Cells are able to respond fast to changing factors in the environment when effects are non-heritable (78). Heritable or long-term epigenetic effects occur in response to long acting stimuli and can be transmitted to memory of the offspring cells (79, 80). PTHMs, along with DNA methylation, microRNAs (miRNAs) and lncRNAs regulate chromosomal function and gene expression.

## Interplay of lncRNAs and Other Epigenetic Mechanisms in Cardiac Fibrosis

With the emergence of genome-wide association studies (GWAS) in the early to late 2000s, nearly 88% of trait/disease-associated single nucleotide polymorphisms were identified to reside in non-protein coding regions (81); which, suggests that alterations in lncRNAs may be implicated in the genetic susceptibility of DCM.

### LncRNAs

LncRNAs are defined as transcripts that are greater than 200 nucleotides in length and do not possess protein-coding potential (82). Based on their genomic localization, lncRNAs can be categorized as sense, anti-sense, bidirectional, enhancer, intronic, and intergenic lncRNAs (82, 83). Moreover, studies within the last decade, have provided unique insights behind the involvement of lncRNAs in a number of biological processes that include genomic imprinting (epigenetic regulation), enhancer activation, scaffold and guide for transcription and epigenetic factors, molecular sponges, and cell-cycle control (4, 82–87). In fact, the localization of the lncRNA transcript can govern its functional capabilities. For example, nuclear lncRNAs can influence gene activation by interacting with chromatin-remodelling complexes that initiates histone or DNA modifications (4, 82–86). Whereas, cytoplasmic lncRNAs are capable of secluding miRNAs (miRs) to indirectly impact protein expressions (4, 82–86).

### LncRNAs and Their Implications in the Heart

**Figure 3:**
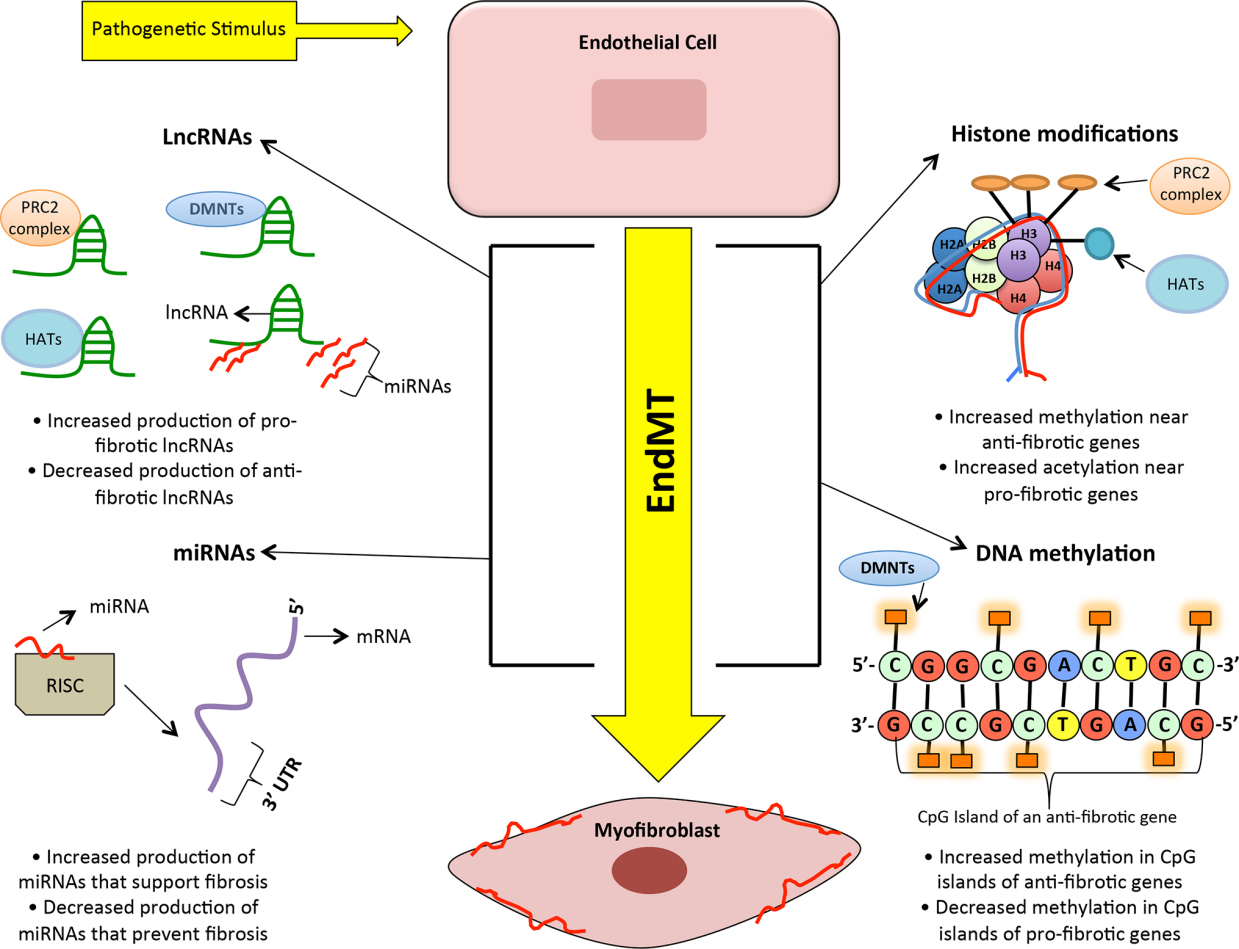
A simplified visual representing the interplay of many epigenetic processes that enhance endothelial-to-mesenchymal transition in diabetic cardiomyopathy. Legend: PRC2 complex = Polycomb repressive complex 2, HDAC = Histone acetyltransferases, DMNTs = DNA methyltransferases, LncRNA = Long non-coding RNAs, miRNA = microRNA, 3’ UTR = three prime untranslated region, mRNA = messenger RNA, and EndMT = endothelial to-mesenchymal transition.

Following the findings from GWAS, recent transcriptomic analyses revealed that lncRNA expression profiles differed considerably between failing and non-failing human hearts (88). In fact, out of the 18,480 total lncRNAs detected in the human heart, nearly 1249 lncRNAs (from ischemic and non-ischemic origins) were differentially expressed with heart failure (88). These findings, along with transcriptomic profiling from other studies (89, 90), opened the door for lncRNA research in cardiac pathologies. To date, there has been a multitude of lncRNAs identified in cardiovascular complications (summarized in Table 1); however, their exact mechanisms in cardiac fibrosis still require further characterization. Nevertheless, emerging studies are beginning to provide insight into some of the roles of lncRNAs in influencing other epigenetic mechanisms such as DNA methylation, histone methylation, histone acetylation and miRs in fibrosis. We will discuss some of the specific lncRNAs and their roles in DCM. However, it is to be noted that lncRNAs can interact and regulate multiple other epigenetic mechanisms such as methylation, acetylation, and miR alterations (shown in Figure 3). A concerted effort of all these pathways ultimately regulates gene expression and increased ECM production. Outlined below is a discussion of these mechanisms and their interactions with lncRNAs.

**Table 1:** List of the prominent lncRNAs implicated in cardiac complications.

**LncRNAs**	**Cells**	**Role in Cardiac Complications**	**Ref**
*Chaer*	EFs*, VMs*, and HT*	Induces cardiac hypertrophy and CD*	(91)
*Mhrt*	Cardiomyocytes	Protects heart from hypertrophy and failure	(92)
*MIAT*	Cardiac Fibroblasts	Induces cardiac fibrosis and sponges miR-24	(93)
*MALAT1*	LVT*	Regulates inflammatory cytokines	(94)
*CHRF*	Cardiomyocytes	Regulates cardiac hypertrophy	(95)
*ROR*	Cardiomyocytes	Promotes cardiac hypertrophy	(96)
*H19*	Cardiomyocytes	Negative regulator of cardiac hypertrophy	(97)
*LIPCAR*	Plasma	Associated with heart failure	(98)
*ANRIL*	PBTL*	Correlates with atherosclerosis risk	(99)
*CARL*	Cardiomyocytes	Prevents mitochondrial fission and sponges miR-539	(100)
*NRF*	Cardiomyocytes	Regulate necrosis and sponges miR-873	(101)
*Wisper*	Cardiac Fibroblasts	Controls cardiac remodeling and fibrosis	(102)
*PRL*	CFs and CMs	Induces cardiac fibrosis and sponges let-7d	(103)

PBTL*, Peripheral blood T-lymphocyte; CFs, Cardiac Fibroblasts; CMs, Cardiomyocytes; LVT*, Left ventricular tissues; EFs*, Embryonic fibroblasts; VMs*, Ventricular myocytes; HT*, Heart tissues; CD*, Cardiac dysfunction.

### DNA Methylation

DNA methylation is a critical epigenetic mechanism that involves the interaction between DNA methyltransferases (DMNTs) and DNA demethylases. Typically, gene silencing, via the elevated activity of DMNTs, is associated with aberrant methylation patterns of CpG dinucleotide clusters (CpG islands) in certain genes (104). In the context of cardiac fibrosis, Watson and colleagues have previously demonstrated *in vitro* that global DNA hypermethylation and elevated activities of DMNTs (DNMT1 and DMNT3B) were associated with hypoxic and pro-fibrotic human cardiac fibroblasts (105). In fact, silencing DMNT3B by small interfering RNAs (siRNAs) or administering the pan-DMNT inhibitor, 5-aza-2’-deoxycytidine, resulted in significant downregulations of α-SMA, collagen 1, and subsequently suppressed the pro-fibrotic effects of TGF-β (105). On the other hand, Pan et al. specifically analyzed CpG sites in the collagen type 1 alpha 1 chain (COL1A1) promoter of rat cardiac fibroblasts following TGF-β treatment and observed significant reductions in DNA methylation and DMNT activity (106). They conclude that TGF-β is capable of promoting collagen type I expression through inhibition of DMNTs in the COL1A1 promoter of cardiac fibroblasts. Moreover, Xu et al. have previously demonstrated that TGF-β also evokes aberrant methylation patterns in the promoter of the Ras-GTPase RASAL1, which in turn hinders the expression of RASAL1 (107). The subsequent reduction in RASAL1 expression allows for heightened Ras-GTP activity, which enhances EndMT and contributes to cardiac fibrosis (107). Xu and colleagues have also documented similar observations in fibrotic cardiac tissues from patients and mice (107, 108). Overall, these findings suggest that pathological stimuli can impact overall DNA methylation activity and ultimately influence the activation of pro-fibrotic genes in cardiac fibrosis.

Although the link between DNA methyltransferases and lncRNAs in cardiac fibrosis has not been made clear, previous evidence in lung fibrosis suggests that miRs (a group of small non-coding RNAs; sncRNAs) exhibit a complex regulatory relationship with DMNTs (109). In fact, Dakhlallah and colleagues revealed that several miRNAs from the miR-17 ~92 cluster targeted DNMT-1 expression and this ultimately produced a negative feedback loop (109). They also identified reduced miR-17 ~92 expressions, and increased DNMT-1 expression and promoter methylation of miR-17 ~92 in fibrotic lung tissues. Furthermore, when looking at lncRNAs, there are several evidences in neural differentiation, skeletal myoblast differentiation, colon cancer, and somatic cell reprogramming that document the capability of lncRNAs to modulate DNA methylation through direct or indirect interactions with various DMNTs (110–113). Whether these mechanistic actions of lncRNAs are present during cardiac fibrosis, requires further exploration.

### Histone Methylation

Histone methylation involves the transfer of methyl groups from the methyl donor S-adenosyl methionine (SAM) to amino acid residues (lysine, arginine and histidine) by histone methyltransferases (114, 115). Methylation at the arginine and lysine residues leads to either activation or repression of transcription. Arginine residue methylation results in only activation, while methylation at lysine can lead to activation or repression of transcription. Generally, H3K4 (histone 3 methylated at lysine 4), H3K36 and H3K79 are associated with transcriptional activation and H3K9, H3K27 and H4K20 are seen in repressed regions(76). To add to the complexity of this regulation, lysine residues can be mono-methylated (m1), di-methylated (m2) or tri-methylated (m3). Furthermore, the specific actions of methyltransferases and demethylases controls chromatin accessibility to transcriptional enzymes and therefore influences protein expression. The arginine and lysine methyltransferase enzymes are part of three protein families: the protein arginine methyltransferase family, the SET-domain-containing protein family of lysine methyltransferases and the non-SET-domain DOTI-like protein (116). A recent study has demonstrated that human ECs transiently exposed to high glucose induced transcriptional activation of NFκB (p65), followed by the overexpression of inflammatory factors. These alterations were attributed to Set7-dependent monomethylation of H3K4 (117, 118). Reversal of oxidative stress through overexpression of ROS-scavenger enzymes prevented both NF-κB activation and vascular inflammation. Hence, oxidative stress plays a critical role in chromatin remodelling and gene alterations during diabetes (117, 118). Furthermore, evidences suggest involvement of important histone modifications in gene regulation that are associated with the pathogenesis of DCM. For example, the H3K79 methyltransferase DOT1L is downregulated in DCM patient samples and may regulate pathologic cardiac remodelling that ultimately contributes to eccentric hypertrophy and reactive fibrosis (119). Our lab has also described regulatory roles of tri-methylation at lysine27 (H3K27me3) catalyzed by H3K27me3 transferase EZH2 (PRC2 component) in diabetic complications (120). EZH2 is part of the polycomb repressive complex 2 (PRC2), which is a multimeric complex known to negatively regulate expression of genes including miRs. Other components of PRC2 are EED, SUZ12 and RpAp46/48 (121). This complex has been widely studied in tumor biology, where increased EZH2 promoted VEGF stimulation and subsequent angiogenesis by inhibitory methylation of anti-angiogenic factor vasohibin1 (vash1) (122). Additionally, Zhu et al. have demonstrated that miR-214–3 p is capable of targeting and reducing the expressions of EZH1 and EZH2, which subsequently attenuated the expressions of Col1a1 and Col3a1 (extracellular matrix genes in cardiac fibrosis) in mice myofibroblasts (123). On the other hand, the enforced expressions of EZH1 and EZH2 significantly contributed to elevated levels of Col1a1 and Col3a1 in myofibroblasts (123). Accumulating evidences in renal and hepatic fibrosis suggests that EZH2 plays a critical role in fibrotic development through the downregulation of PPAR-γ, Smad7 and PTEN to further enhance pathways implicated in profibrotic signalling (124, 125).

Within the last decade, various cancer- and inflammatory-based studies have identified several lncRNAs that bind to chromatin modification complexes, such as PRC2, to regulate gene expression (126–128). In the context of the heart, the lncRNA *Fendrr* is known to bind to both PRC2 and TrxG/MLL complexes in order to facilitate proper cardiac development (129). With this in mind, studies depicting the relationship between lncRNAs and chromatin-remodelling complexes in facilitating cardiac fibrosis are slowly beginning to emerge. For example, the lncRNA *Chaer* (cardiac-hypertrophy-associated epigenetic regulator) directly interacts with EZH2 to alter PRC2 targeting, which ultimately inhibits H3K27me3 at the promoter regions of genes implicated in cardiac hypertrophy (130). Klattenhoff and colleagues demonstrate that the lncRNA *Bvht* interacts with SUZ12 of PRC2 to mediate epigenetic regulation of cardiovascular lineage commitment (131). While, *Mhrt* is a cardioprotective lncRNA that is capable of protecting the heart from hypertrophy and failure by sequestering a stress-induced chromatin-remodelling factor (Brg1)— this factor is then unable to activate aberrant gene expressions (92). Moreover, in the context of lung cancer and epithelial-mesenchymal transition, Terashima and colleagues have shown through chromatin immunoprecipitation that the lncRNA *MEG3* can transcriptionally repress miR-200 family genes by recruiting EZH2, and histone H3 methylation to these specific regulatory regions (132). Whether similar mechanisms exist in EndMT during DCM still remains elusive; however, previous researches have already documented unique lncRNAs in cardiovascular complications: *MIAT* (93), *MALAT1* (94), *CHRF* (133), *ROR* (96), *H19* (97), *LIPCAR* (98) and *ANRIL* (99). Using this information, identifying the chromatin-lncRNA interactions will provide additional mechanistic/functional insight behind these unique lncRNAs in cardiac fibrosis. We have previously shown that *ANRIL* regulates glucose-mediated upregulation of VEGF through its interactions with EZH2 and p300 (a histone acetyltransferase) in glucose-treated ECs (134). Nevertheless, of note, EZH2 can also act as a platform for the recruitment of DMNTs and directly control DNA methylation (135)—which alludes to the importance of understanding epigenetic mechanisms in its entirety.

### Histone Acetylation

Histone acetylation involves either the addition or removal of acetyl groups to lysine residues, which is facilitated by histone acetyltransferases (HATs) and histone deacetylases (HDACs), respectively (136, 137). The interplay between HATs and HDACs can govern gene regulation in which elevated acetylation at specific lysine residues (lysines 9, 14, 18 and 56 in histone H3 and lysines 5, 8, 13 and 16 in histone H4) allows for chromatin relaxation and heightened transcription factor recruitment—contributing to gene activation (70, 138). As well, HATs and HDACs can also directly modify regulatory proteins and transcription factors (138). Previous work by our lab demonstrated that the transcriptional coactivator and HAT, p300 was markedly expressed in the diabetic animal heart and an increase in p300 activity led to an upregulation of VEGF, endothelin-1 (ET-1), and FN—molecules that are implicated in DCM (139, 140). In fact, silencing p300 prevented the diabetes-induced expression of VEGF, ET-1, and FN (139, 140). Our findings also demonstrated that p300 had an increased binding to ET-1 and FN promoters in large vessel ECs cultured with high glucose, which was also associated with augmented histone acetylation, H2AX phosphorylation, activation of several transcription factors, and mRNA expressions of ECM proteins and vasoactive factors (139). Similarly, Ghosh and colleagues have further indicated that p300 is an essential coactivator of pro-fibrotic signalling and is dramatically elevated during cardiac EndMT (141). Moreover, in the context of histone deacetylases, silent mating type information regulation 2 homolog (SIRTs) are important NAD-dependent deacetylases that are involved in a number of cellular processes such as apoptosis, and fatty acid and glucose metabolism (142–144). *In vitro* and *in vivo* findings from our lab have previously demonstrated that SIRT1 (a type III HDAC) activity is significantly reduced under chronic hyperglycemic environments in ECs and the subsequent reduction of SIRT1 drove the formation of reactive oxygen species (ROS), which is mediated by FOXO1(forkhead box protein O1) acetylation through elevated p300 activity (145). SIRT1 is also capable of regulating TGF-β1 and ET-1 expressions by p300, while SIRT1 overexpression can prevent diabetes-induced FN upregulation (146). Rizk and colleagues have also shown similar findings where elevating SIRT1 levels, with the administration of L-arginine, significantly prevented diabetes-induced myocardial fibrosis in male Wistar rats (146). Along with their histopathological findings, Rizk and colleagues observed a reduction in the expression of cardiac fibrotic markers (FN, TGF-β, brain naturetic peptide, and connective tissue growth factor) following SIRT1 upregulation (146). Other studies are also in agreement that SIRT1 activation protects against cardiovascular damage (147–149).

Although lncRNAs such as *ANRIL* (134) and *Khps1* (150) have been shown to interact with p300 in microvascular ECs and cancer cell lines, respectively, this form of interaction has not yet been documented in cardiac fibrosis. However, our lab has previously identified that miR-200b (a sncRNA) is capable of mediating EndMT and VEGF through direct targeting of p300 in heart and retinal tissues of diabetic mice and rats (151–153). Additional work by Shehadeh and colleagues have reported the ability of miR-20a to bind to the 3’ untranslated region (UTR) of p300 and directly repress the expression of p300—subsequently reducing pro-angiogenic gene expressions implicated in cardiac hypertrophy (154). As for interactions between SIRTs and lncRNAs, recent evidence suggests that the *Sirt1 antisense* (AS) lncRNA can inhibit muscle formation by activating *Sirt1* and impeding the function of miR-34a by competitively interacting with the 3’ UTR of the *Sirt1* transcript (130). On a similar note, several miRs that are capable of modulating SIRT1 activity have been documented previously (155). Nevertheless, further follow-up is required for the role of *Sirt1 AS* lncRNA in cardiac fibrosis.

### miRNAs (MiRs)

As previously mentioned, miRs belong to the class of small non-coding RNAs that are ~20–25 nucleotides in size (156, 157). MiRs are synthesized by RNA polymerase II, processed to precursors in the nucleus by RNAse III Drosha and DiGeorge syndrome critical region 8 (DGCR8) and then exported into cytoplasm by exportin 5 (157, 158). Dicer further processes miRs in the cytoplasm into functionally active miRNAs (159). From there, with the help of argonaute proteins, active miRNAs are incorporated into the RNA-induced silencing complex (RISC) (157–159). Following the complete formation of RISC, miRs are then capable of binding to complementary sequences within the 3’ UTR of targeted mRNAs, which subjects these transcripts to degradation or repressed translation—ultimately, inhibiting protein expression (157–159). From our lab, we have previously reported hyperglycemia-induced alterations of miRs in ECs and in numerous tissues impacted by chronic diabetes: miR-1, miR-133a, miR-146a, miR-195, miR-200b and miR-320 (120, 152, 153, 160–164). Earlier findings from our previous reports also demonstrate a novel regulation mechanism between PRC2 and miRNAs through histone methylation in diabetic complications (120, 153). As well, we show that the repression of miRs-146a and 200b plays an integral role in enhancing glucose-induced EndMT (152, 162). Moreover, within the last decade, a multitude of studies have emerged that investigate the regulatory capabilities of miRNAs in diabetes and its complications (165–167), and explore the changes of miRNAs upon various treatments (168–170). In the context of the heart, miRs-1, 22, 29, 31 101, 133, and 489 are few examples of the many miRs that may be functioning as inhibitors of cardiac fibrosis and hypertrophy (133, 171–175). Whereas, miRs- 21, 34, 132, 199b, 208a and 212 have been reported to enhance cardiac complications (87, 176–180).

One of the important functions of lncRNAs is to act as a molecular sponge to certain miRs, which can ultimately hinder the expression of these small non-coding molecules (181). For instance, the cardiac-apoptosis related lncRNA (*CARL*) has been shown to block the actions of miR-539 by acting as an endogenous sponge that consequently augments the expression of PHB2 (a miR-539 target) and prevents cardiomyocyte apoptosis and mitochondrial fission (133). Similarly, the lncRNA *NRF* (necrosis-related factor) can regulate necrosis in cardiomyocytes by sponging miR-873, which is an important miR implicated in the translational repression of RIPK1/RIPK3 (130). Furthermore, in cardiac fibroblasts, *MIAT* (myocardial infarction associated transcript) can block the actions of miR-24 (a critical regulator in TGF-β1 activation) by its sponging capabilities, and induce cardiac fibrosis (182). The findings from the study by Qu and colleagues identified and characterized *MIAT* as the first profibrotic lncRNA involved in cardiac fibrosis. Moreover, a recent study by Liang et al. has demonstrated that the pro-fibrotic lncRNA (PFL) can act as a competitive endogenous RNA for miR let-7d (103). Specifically, the overexpression of PFL stimulated proliferation, fibroblast-myofibroblast transition and fibrogenesis in mice cardiac fibroblasts by reducing the activity and expression of let-7D (103). Micheletti and colleagues have also recently shown that the lncRNA *Wisper* can regulate cardiac remodeling and fibrosis; however, whether *Wisper* can interact with miRs remains to be determined (102). Additional characterization of other profibrotic lncRNAs will provide significant insight behind the pathogenetic mechanisms of cardiac pathologies. Therefore, establishing a database with the fibrotic capabilities of previously documented lncRNAs in other diseases could help achieve this. For example, in the context of liver fibrosis, *Hotair* has been shown to act as an endogenous sponge for miR-148b to facilitate DMNT1 expression (183). Further follow-up on the sponging capabilities of *Hotair* in a cardiac-specific context may provide important information behind the precise nature of these regulatory mechanisms behind fibrosis and other cardiac complications.

## Future Direction

Our review summarized the key epigenetic mechanisms implicated in DCM fibrosis; as well, we have provided unique insights behind lncRNAs and their impact on DNA methylation, histone modifications, and miRs. Although direct evidence for the mechanisms of lncRNAs is still limited in the context of cardiac fibrosis, emerging studies are beginning to provide further understanding of the regulatory capabilities of lncRNAs during DCM. Moreover, the critical implications of lncRNAs in a multitude of biological processes make it a valuable target for therapeutic applications, which necessitates the need for additional research behind lncRNA and epigenetic protein interactions. Therefore, understanding these epigenetic mechanisms for lncRNAs in cardiac fibrosis will not only provide critical information behind the intricate gene network, but it will also facilitate the development of better-targeted treatment strategies that take into account the regulatory gene network in its entirety. We hope that our review will allow for critical discussions and further experimentation that will extend the current findings of these epigenetic mechanisms on lncRNAs during cardiac fibrosis in DCM.

## Data statement

The figures and table presented in this review were drawn by Saumik Biswas and Subrata Chakrabarti. We provide permission to *Frontiers *to publish our illustration in all formats (i.e. print and digital). 

## Author Contributions

Writing of manuscript: SB, AT, SC. Figure drawings: SB, SC.

## Conflict of Interest Statement

The authors declare that the research was conducted in the absence of any commercial or financial relationships that could be construed as a potential conflict of interest.
